# Cross-Sectional Associations between Dietary Daily Nicotinamide Intake and Patient-Reported Outcomes in Colorectal Cancer Survivors, 2 to 10 Years Post-Diagnosis

**DOI:** 10.3390/nu13113707

**Published:** 2021-10-21

**Authors:** Wenbo Wu, Martijn J. L. Bours, Annaleen Koole, Marlou-Floor Kenkhuis, Simone J. P. M. Eussen, Stephanie O. Breukink, Frederik-Jan van Schooten, Matty P. Weijenberg, Geja J. Hageman

**Affiliations:** 1 Department of Pharmacology and Toxicology, Maastricht University, 6200 MD Maastricht, The Netherlands; f.vanschooten@maastrichtuniversity.nl (F.-J.v.S.); g.hageman@maastrichtuniversity.nl (G.J.H.); 2 NUTRIM School for Nutrition and Translational Research in Metabolism, Maastricht University, 6200 MD Maastricht, The Netherlands; s.breukink@mumc.nl; 3 Department of Epidemiology, Maastricht University, 6200 MD Maastricht, The Netherlands; m.bours@maastrichtuniversity.nl (M.J.L.B.); annaleen.koole@maastrichtuniversity.nl (A.K.); m.kenkhuis@maastrichtuniversity.nl (M.-F.K.); simone.eussen@maastrichtuniversity.nl (S.J.P.M.E.); mp.weijenberg@maastrichtuniversity.nl (M.P.W.); 4 GROW School for Oncology and Developmental Biology, Maastricht University, 6200 MD Maastricht, The Netherlands; 5 CARIM School for Cardiovascular Diseases, Maastricht University, 6200 MD Maastricht, The Netherlands; 6 CAPHRI School for Care and Public Health Research Institute, Maastricht University, 6200 MD Maastricht, The Netherlands; 7 Department of Surgery, Maastricht University Medical Centre+, 6200 MD Maastricht, The Netherlands

**Keywords:** colorectal cancer survivor, fatigue, patient-reported outcomes, nicotinamide, NAD^+^ precursor

## Abstract

Supplementation with nicotinamide adenine dinucleotide (NAD^+^) precursors including dietary nicotinamide has been found to boost tissue NAD^+^ levels and ameliorate oxidative stress-induced damage that contributes to aging and aging-related diseases. The association between dietary NAD^+^ precursors and patient-reported health-related outcomes in cancer survivors has not been investigated. This study aimed to determine associations of dietary nicotinamide intake with different patient-reported outcomes in colorectal cancer survivors, 2 to 10 years post-diagnosis. A total of 145 eligible participants were recruited into this cross-sectional study. Dietary nicotinamide intake level was calculated based on data from 7-day food diaries. Fatigue was assessed with the Checklist Individual Strength (CIS), which is a subscale of the cancer-specific European Organization for the Research and Treatment of Cancer Quality of Life Questionnaire-Core 30 (EORTC), and anxiety and depression were assessed with Hospital Anxiety and Depression Scale (HADS). Oxidative stress marker serum protein carbonyl contents and serum NAD^+^ levels were measured. A hierarchical linear regression model with confounder adjustment was performed to analyze the association of nicotinamide intake, serum protein carbonyl contents, and NAD^+^ levels with patient-reported outcomes. The median values of daily nicotinamide intake for male and female participants were 19.1 and 14.4 mg, respectively. Daily dietary nicotinamide intake was associated with a lower level of fatigue (β: −14.85 (−28.14, −1.56)) and a lower level of anxiety and depression (β: −4.69 (−8.55, −0.83)). Subgroup analyses by sex showed that a beneficial association between nicotinamide intake and patient-reported outcomes was mainly found in men. To conclude, our findings suggested that higher dietary NAD^+^ precursor nicotinamide intake was cross-sectionally associated with less patient-reported outcomes in CRC survivors.

## 1. Introduction

Colorectal cancer (CRC) is the third-most common cancer worldwide in men and women combined [1]. The incidence of CRC is expected to rise up to 2.2 million new cases per year by 2030 [2]. Increased aging of the population, unfavorable dietary habits, and specific risk factors (e.g., overweight, physical inactivity and sedentary behavior, alcohol consumption, smoking, and comorbidities such as inflammatory bowel disease and type II diabetes mellitus) have been reported to account for this trend in CRC incidence [3]. At the same time, early detection and improved treatments have improved the CRC survival rates in recent decades [4]. CRC survivors often report various complaints or adverse effects after treatment, such as fatigue, pain, impaired sleep, and mental problems, which may affect health-related quality of life (HRQoL) [5]. Thus, identifying targets and factors for interventions to promote better HRQoL of CRC survivors is warranted.

As mentioned, aging is a critical factor contributing to the growing population of CRC survivors. Aging is marked by a systemic decrease in nicotinamide adenine dinucleotide (NAD^+^) across multiple tissues [6]. NAD^+^ declines have been linked with aging and aging-related disorders, including age-associated metabolic disorders, cancer, neurodegenerative diseases, and mental disorders [7,8]. NAD^+^ is a co-enzyme involved in cellular metabolism and has a regulating role in various biological processes. Its biosynthesis and turnover have recently been attracting more interest [7,9]. When the activities of NAD^+^-dependent enzymes, such as CD38/157 and poly (ADP-ribose) polymerase-1 (PARP-1) are chronically increased, specifically under chronic inflammation and oxidative stress, this may lead to NAD^+^ decline and a reduced substrate availability for sirtuins that are involved in anti-aging signaling [8]. Supplementation with small molecular compounds used for NAD^+^ biosynthesis, so-called NAD^+^ precursors, such as nicotinamide riboside (NR), nicotinamide mononucleotide (NMN), and nicotinamide (NA), has been found to exert preventive and therapeutic effects that ameliorated aging-associated pathophysiologies in animal models [10,11,12,13]. These emerging findings have brought a promising intervention, a so-called “NAD^+^ boosting” strategy. A recent study showed that supplementing patients with niacin (nicotinic acid), a dietary NAD^+^ precursor, indeed ameliorated NAD^+^ deficiency and improved muscle performance in patients with adult-onset mitochondrial myopathy [14].

CRC survivors frequently report fatigue, depression, and anxiety, and yet the underlying mechanisms remain elusive. Cancer treatment, especially chemotherapy and radiotherapy, have previously been found to be associated with impaired health outcomes [15]. Inflammation and oxidative stress triggered by chemotherapeutic agents can be a double-edged sword. On the one hand, when inducing treatment response against tumor cells, it can also impair normal cells. On the other hand, chemotherapeutic agents can induce cell senescence, which could lead to a senescence-associated secretory phenotype (SASP), a complex panel of excreted factors that may bring chronic systemic inflammation and contribute to increased NAD^+^ consumption [16]. Evidence for an ameliorating effect of a NAD^+^ boosting strategy was obtained in a recent animal study. When a diet supplemented with nicotinamide, a form of Vitamin B3 and the most common NAD^+^ precursor, was fed to mice, it was found to reduce oxidative stress and inflammation, maintain NAD^+^ levels, and improve health [13]. Even though nicotinamide-rich foods are widely available and nicotinamide intake in the general Dutch population is generally meeting the requirements [17], chronic inflammation occurs in CRC survivors [8,18,19] and may contribute to an increased degradation of NAD^+^, resulting in a disruption of NAD^+^ homeostasis. However, whether and how a disturbed NAD^+^ homeostasis may be related to patient-reported outcomes in CRC survivors has not been investigated. Therefore, in this cross-sectional study with CRC survivors 2 to 10 years after diagnosis, we aimed to investigate how dietary nicotinamide intake, serum NAD^+^ concentration (as a marker of NAD^+^ status), and protein carbonyl contents (as a marker for oxidative stress) are associated with hand-grip strength and patient-reported outcomes including emotional and cognitive functioning, fatigue, depression, and anxiety.

## 2. Materials and Methods

### 2.1. Study Design and Population

**Figure 1:**
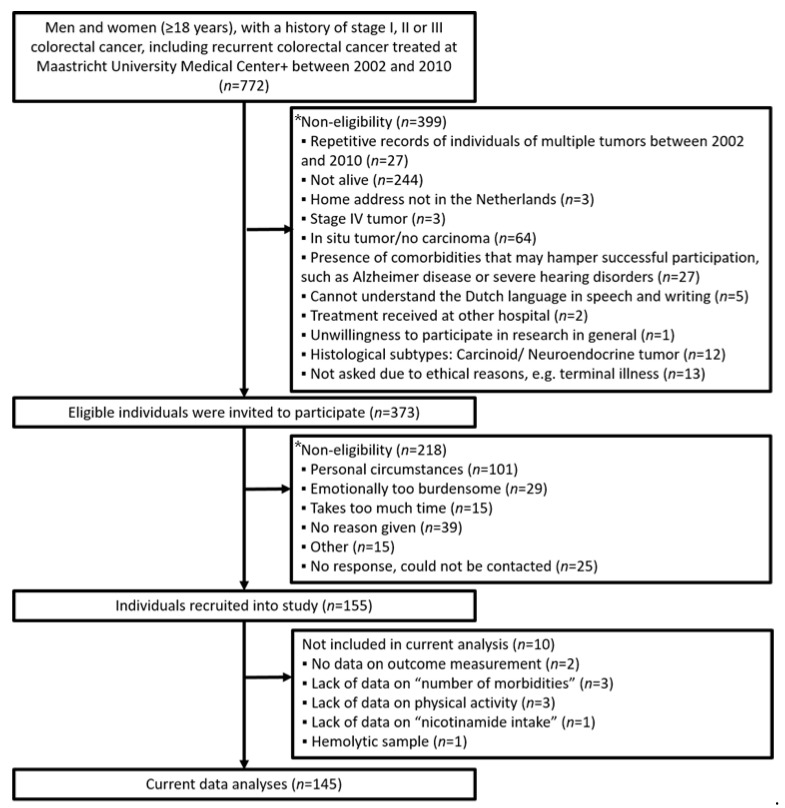
Flow diagram of individuals included in the cross-sectional part of the EnCoRe study (*: some participants may have multiple reasons).

Data from the “Energy for Life after ColoRectal Cancer” (EnCoRe) study were used, which has been described previously [20]. The EnCoRe study includes an ongoing prospective part and a cross-sectional part. Data from the cross-sectional part were used for the present analyses. Basically, the cross-sectional study included CRC survivors 2 to 10 years after diagnosis of stage I to III CRC. Eligible participants who were diagnosed and treated between 2002 and 2010 at Maastricht University Medical Center+, the Netherlands, were recruited into the study between 2012 and 2013. An overview of patient recruitment including reasons for exclusion is given in Figure 1; in total, 145 subjects were eligible for the current study. The EnCoRe study has been approved by the Medical Ethics Committee of the Academic Hospital Maastricht and Maastricht University, the Netherlands (Project identification code: METC 11-3-075). Informed consent was signed by all participants.

### 2.2. Calculation of Average Daily Nicotinamide Intake

A seven-day dietary record was obtained from each participant to quantitatively estimate food intake. All subjects received detailed written and oral instructions during a home visit for determining the type and amount of food to be recorded. The dietary record contained three standard mealtimes (breakfast, lunch, and dinner) and any snack moments in between. Participants were requested to provide a sufficient amount of detail, such as the brand names of consumed foods, ingredients used, quantified food intake (using either grams or standardized household measures, i.e., tablespoon, glass, etc.), cooking methods (e.g., boiled, in oven, or marinade), and recipes (including ingredients for ready-to-serve meals). Upon receipt of completed dietary records, a quality control check for consistency and completeness was conducted afterwards by the researchers. In case of any inconsistencies or missing information, participants were contacted for additional information. Next, dietary records were coded by trained dieticians based on the Dutch Food Composition Database 2011 (NEVO), which contains energy and macro- and micro-nutrient values for each food and drink. More details were given previously [21,22,23]. Average daily nicotinamide intake was calculated in milligrams per day by multiplying food frequencies and dosage. In addition to the dietary records, information on the use of dietary supplements was collected according to the procedures described previously [22]. Briefly, participants were asked to report whether one or more supplement was used, the start and stop date, the ingredients, and the motivations for use. Each participant was also asked to provide the original package of the supplements if available.

### 2.3. Measurement of Serum Protein Carbonyl Contents and NAD^+^ Levels

A venous blood sample was drawn from study participants during a home visit. Blood samples were collected in 8.5 mL serum tubes (BD Vacutainer SST II Advance), and after centrifugation, aliquots were cryopreserved at −80 °C within 4 h after blood drawing until analysis. Serum concentrations of protein carbonyl products were measured based on 2,4-dinitrophenylhydrazine (DNPH) colorimetric assay [24]. The concentration of protein carbonyl products was normalized using the total protein concentration of serum, which was determined with the Bradford assay. Serum NAD^+^ levels were determined spectrophotometrically using the thiazolyl blue cycling assay established by Bernofsky and Swan [25]. Briefly, each assay contained 100 mM bicine, pH 7.8; 500 mM ethanol; 0.42 mM 3-[-4,5-dimethylthiazol-2-yl]-2,5-diphenyl tetrazolium bromide (MTT); 1.66 mM phenazine methosulfate (PMS); and 14 units alcohol dehydrogenase (ADH). The amounts of NAD^+^ were measured as the change in absorbance at 590 nm at 37 °C for 10 min with a Model 680XR microplate reader (BioRad, Hercules). In case of potential contamination of erythrocytes caused by blood drawing, a quality control procedure by measuring serum free hemoglobin levels (fHB) determined by 3,3′,5,5′-tetramethylbenzidine (TMB) assay was also carried out [26]. The cut-off value for hemolytic samples was set on fHB ≥ 1.4 mg/mL [27,28]. The hemoglobin concentrations presented in the serum sample were relatively low (below 0.059 mg/mL), indicating that the contamination of potential erythrocytes during sample collecting can be negligible. Quality control (QC) samples were introduced, and both study and QC samples were analyzed in triplicate. Coefficient of variation (CV) within (inter-assay CV) and between different runs (intra-assay CV) were calculated, and both the inter and intra-assay CV for the measurements were below 10%.

### 2.4. Outcome Measurement

Fatigue was measured by the Checklist Individual Strength (CIS), which is a 20-item questionnaire measuring complaints of fatigue on a 7-point Likert scale. The CIS contains four subscales on separate dimensions of fatigue, i.e., subjective feelings of fatigue (scores ranging from 8 to 56), concentration problems (scores ranging from 5 to 35), reduced motivation (scores ranging from 4 to 28), and reduced physical activity (scores ranging from 3 to 21). Then, scores of individual CIS subscales were summed to a total score for fatigue, ranging from 20 to 140 [29,30,31,32]. Higher scores indicate more severe levels of fatigue.

The 14-item Hospital Anxiety and Depression Scale (HADS) was used to assess levels of psychological distress (scores ranging from 0 to 42), including an anxiety and a depression subscale. Scoring for each item ranges from zero to three, with three denoting highest anxiety or depression level. Fatigue, emotional, and cognitive functioning were addressed by specific subscales of the cancer-specific European Organization for the Research and Treatment of Cancer Quality of Life Questionnaire-Core 30 (EORTC QLQ-C30, version 3.0) [31]. Item scores were each converted to a scale ranging from 0 to 100, with higher scale scores representing more fatigue or better functioning.

Dominant hand maximum isometric hand-grip strength, representing muscle function, was measured with a Jamar hand dynamometer (Sammons Preston Rolyan, Bolingbrook, IL, US). Two measurements were conducted per participant, of which the highest value was used as maximum hand-grip strength [31].

### 2.5. Other Factors

Information on demographic factors (age, sex) and smoking status were obtained through self-report. Comorbidities were assessed by the Self-Administered Comorbidities Questionnaire [20,30]. Clinical characteristics such as date of CRC diagnosis, tumor stage, and treatment(s) received (chemotherapy, radiotherapy, and/or surgery) were obtained through the Netherlands Cancer Registry. Body height and weight, to calculate body mass index (BMI in kg/m^2^), were measured by trained research personnel during a home visit. The “Short QUestionnaire to ASsess Health enhancing physical activity (SQUASH)” was used to measure the level and intensity of physical activity by assessing the frequency (days per week), duration (time per day), and intensity (low, moderate, high) of different types of activities [20]. Moderate-to-vigorous physical activity (MVPA, hours/week) was regarded as all activity of more than 3 metabolic equivalents (MET) [20,33].

### 2.6. Statistical Analysis

Descriptive analysis on demographic and clinical characteristics was performed using means and standard deviations (SD) for normally distributed continuous variables or medians and interquartile ranges (IQR) for non-normally distributed variables. Frequencies and percentages were used to describe categorical variables. Due to the right-skewed distribution of nicotinamide intake, a natural-log transformation was done to obtain a normal distribution. Linear correlation between daily nicotinamide intake with natural-log transformation and the other variables (including all the biomarkers, i.e., serum NAD^+^ levels and protein carbonyl contents) was assessed with Pearson or Spearman correlation coefficients based on the type and distribution of variables.

To assess the relation of nicotinamide intake, serum NAD^+^ levels, and protein carbonyl contents with the outcomes of interest, i.e., hand-grip strength, emotional and cognitive functioning, fatigue, and anxiety and depression, multivariate linear regression models with a hierarchical approach were used to estimate unstandardized regression coefficients (β) with 95% confidence intervals (CIs). βs represent the confounder-adjusted difference in score of the outcome per one-unit increase in the Ln-transformed value of the average daily nicotinamide intake as the independent variable. Relevant confounders that were included in all models based on evidence from the literature and biological plausibility were sex, age (years), BMI (kg/m^2^), daily energy intake (kcal), number of comorbidities (0/1/2+), and treatment with chemotherapy (yes/no). Additionally, further potential confounders were included based on whether inclusion of the variable changed the adjusted beta-coefficient of interest by 10% or more. These additional covariates included supplement use (yes/no) and MVPA (hours/week). According to National Institutes of Health (NIH), the recommended dietary allowance (RDA) of nicotinamide intake is different between male and female adults; i.e., for male adults, it is 16 mg/d, while it is 14 mg/d for female adults [34]. Therefore, stratified analysis by sex was performed, and the independent variable was dichotomized into two categories based on recommended RDA for nicotinamide (meet the RDA or not). This allows for better understanding of association between sex-specific nicotinamide intake levels and outcomes. In addition, associations between outcomes and nicotinamide intake dosage (below RDA, between 1 and 1.25 times of RDA, above 1.25 times of RDA) were analyzed to investigate a dose-response relation. The cut-off intake level used for the dose-response relation was set based on the sample numbers and were similar within each dichotomized group. The *p* value for linearity trend was calculated by including the dichotomized groups as a continuous variable in the model. Analyses were performed using IBM SPSS Statistics (Version 25, IBM Corporation: Armonk, NY, USA); statistical significance was set at *p* < 0.05 (two-sided).

## 3. Results

### 3.1. Participant Characteristics

A total of 145 participants were included in the present analyses (Figure 1), of which 62.8% were males (Table 1). The mean (±SD) age was 70.0 ± 9.0 years. The median (IQR) time since diagnosis was 6.0 (3.0) years, and the majority of the participants had received chemotherapy (51.7%), radiotherapy (37.2%), and surgery (95.9%). The median values (IQR) of daily nicotinamide intake for male and female participants were 19.1 (7.5) and 14.4 (6.3) mg, respectively.

Negative correlations were observed for dietary nicotinamide intake with age (Pearson *r* = −0.303, *p* < 0.05) and with the number of comorbidities (Spearman *rho* = −0.314, *p* < 0.05). Positive correlations were observed with MVPA (Pearson *r* = 0.268, *p* < 0.05), sex (Spearman *rho* = 0.509, *p* < 0.05), smoking status (Spearman *rho* = 0.211, *p* < 0.05), and average daily energy intake (Pearson *r* = 0.692, *p* < 0.05). No significant correlations were found with the other variables, including the biomarkers serum protein carbonyl contents and plasma NAD^+^ concentrations (data are not shown).

### 3.2. Associations between Dietary Nicotinamide Intake and Patient-Reported Outcomes

As shown in Table 2, daily nicotinamide intake was significantly associated with hand-grip strength, emotional functioning, activity, and motivation subscale of the CIS questionnaire in the univariate model and model I, while in the fully adjusted model, no significance associations were observed. In fully adjusted models, as shown in Table 2, daily nicotinamide intake was significantly and inversely associated with EORTC fatigue subscale (β: −14.85, 95% CI: −28.14, −1.56), CIS total fatigue (β: −17.52, 95% CI: −34.54, −0.51), CIS subjective fatigue (β: −10.47, 95% CI: −18.70, −2.25), HADS total distress score (β: −4.69, 95% CI: −8.55, −0.83), and HADS anxiety score (β: −2.55, 95% CI: −4.79, −0.32). A 10% higher daily dietary nicotinamide intake (per mg) was associated with a score 34 points lower on the EORTC fatigue scale that ranges from 0 to 100. No statistically significant associations were observed for daily dietary nicotinamide with the cognitive functioning and concentration subscale of the CIS questionnaire in all models.

**Figure 2:**
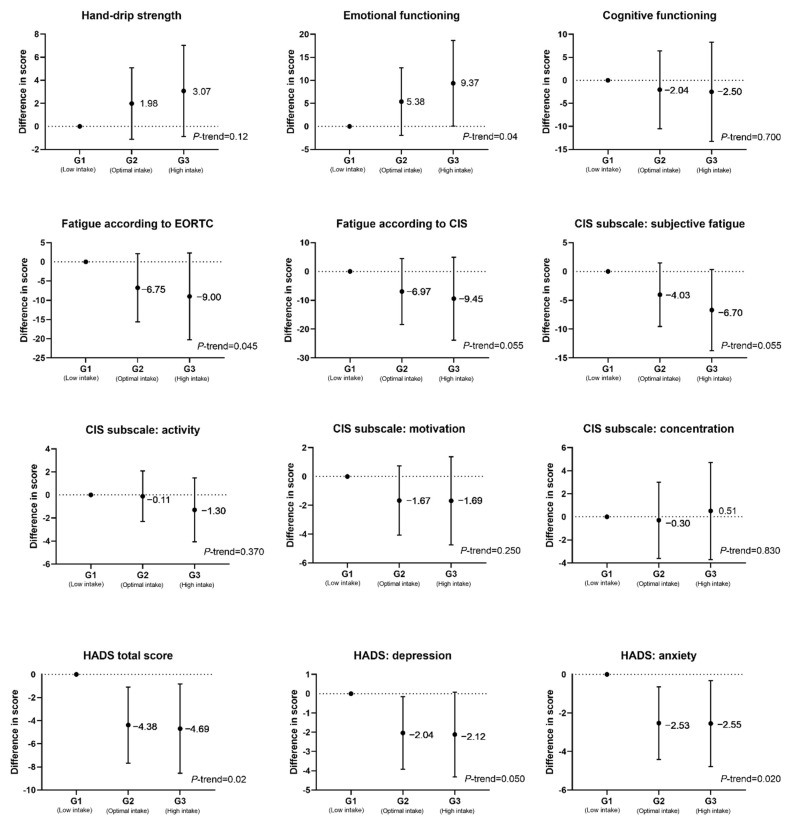
Dose-response relationships between daily nicotinamide intake and hand-grip strength, emotional and cognitive functioning, fatigue, anxiety, and depression. Nicotinamide intake was dichotomized into three groups, G1 (low intake level: below RDA; for male participants, it is <16 mg/day, while for female participants, it is <14 mg/day); G2 (optimal intake level: between RDA and 1.25 times RDA; for male and female participants, 16–20 mg/day and 14–17.5 mg/day, respectively); and G3 (high intake level: above 1.25 times of RDA, >20 mg/day and 17.5 mg/day for male and female participants, respectively). Abbreviations: EORTC, European Organization for the Research and Treatment of Cancer Quality of Life Questionnaire; CIS, Checklist Individual Strength; HADS, Hospital Anxiety and Depression Scale; RDA, recommended dietary allowances.

When the daily nicotinamide intake was dichotomized according to the RDA for men and women, respectively, sex-stratified analyses showed that only the associations observed in male participants were statistically significant (Table 3). In men, nicotinamide intake above the RDA was associated with a significantly lower levels of fatigue, as assessed by both the EORTC (β: −10.43, 95% CI: −20.93, −0.17) and CIS total (β: −8.97, 95% CI: −23.14, −0.20) questionnaires, a better emotional functioning (β: 11.21, 95% CI: 1.64, 20.79), and a significantly reduced level of total depression and anxiety (β: −3.50, 95% CI: −6.94, −0.50), and these significant associations were not observed in women. Significant interaction between sex and nicotinamide intake was found in emotional functioning (*P*-interaction = 0.002), EORTC fatigue (*P*-interaction = 0.005), and HADS anxiety score (*P*-interaction = 0.005). Significant dose-response associations were observed in emotional functioning and HADS total scores when comparing the scores of participants having high (>1.25 RDA, i.e., >20 mg/d for male participants and >17.5 mg/d for female participants) and moderate nicotinamide intake levels (between 1 and 1.25 RDA, i.e., 16–20 mg/d for male participants and 14–17.5 mg/d for female participants), with participants having lower intake levels (<16 mg/d for male participants and <14 mg/d for female participants; Figure 2 and Supplementary Table S1).

In addition to nicotinamide intake, we also analyzed associations between the biomarkers serum NAD^+^ levels, protein carbonyl contents, and patient-reported outcomes, but these were not associated with any of the outcome parameters (Supplementary Tables S2 and S3).

## 4. Discussion

To the best of our knowledge, this is the first study to investigate cross-sectional associations between dietary nicotinamide intake and patient-reported outcomes, i.e., fatigue, anxiety, depression, emotional and cognitive functioning, and hand-grip strength in CRC survivors. Our findings indicate that a higher intake of nicotinamide, the main dietary NAD^+^ precursor, was significantly associated with lower levels of self-reported fatigue and anxiety in CRC survivors 2–10 years post-diagnosis in analyses adjusted for age, sex, BMI, daily average energy, number of comorbidities, received chemotherapy (yes/no), hours of per week MVPA, years since diagnosis, and supplement usage (yes/no). Dose-response associations were observed when modeling nicotinamide intake categorized into three groups based on different intake levels (low, optimal, and high) with fatigue and anxiety and depression. We did not observe significant associations with hand-grip strength or emotional or cognitive functioning after adjusting for relevant confounders.

Complaints of fatigue are frequently reported by CRC survivors [35]. According to current intervention guidelines on fatigue in cancer survivors, physical activity interventions have been found to counteract fatigue [36]. However, due to comorbidities or advanced age, some older adults may not have the required strength for intensive exercise, which may restrict the practical application of such interventions among CRC survivors. Compared to physical activity interventions, nutrition and other supplement interventions are generally tolerable and may have a wider application or may be included in physical intervention programs. Although underlying mechanisms remain elusive, it is generally believed that nutritional status may be an important factor of fatigue. There are only a limited number of human studies covering this field, most of which have found that Vitamin D supplementation and high levels of Vitamin B6 status may be beneficial to alleviate fatigue and other patient-reported outcomes [30,37,38], and in general adhering to specific dietary WCRF/AICR (World Cancer Research Fund and the American Institute for Cancer Research) recommendations is associated with better HRQoL and less fatigue in CRC survivors [39]. However, most evidence for an association between dietary components and fatigue has been derived from studies with patients suffering from chronic fatigue syndrome (CFS). In these studies, nutritional deficiencies of Vitamin C, Vitamin B, sodium, magnesium, zinc, folic acid, L-carnitine, L-tryptophan, essential fatty acids, and co-enzyme Q10 have been reported in CFS subjects [40,41]. As for depression, stress, and anxiety syndromes, earlier studies have mostly been targeted on the general populations instead of CRC survivors. Dietary B vitamins intake was found to be associated with decreased prevalence of depression, anxiety, and stress syndromes [42,43]. More specifically, higher intakes of vitamin B6 [44], folate [44,45], B12 [46], and biotin [47] were negatively associated with psychological distress. Our findings add to the findings of these reports that documented the association between dietary vitamin B intake and distress, since nicotinamide is one of the forms of vitamin B3.

As an essential micronutrient, nicotinamide is also serving as one of the NAD^+^ generating dietary precursors [8]. Supplementing NAD^+^ generating precursors has been found effective in animal studies to counteract oxidative stress and chronic inflammation, which are associated with aging and aging-related diseases. Nicotinamide supplementation reduced oxidative stress and inflammation in high-fat diet obese mice [48]; nicotinamide mononucleotide supplementation reversed vascular dysfunction, reduced oxidative stress, and restored SIRT 1 activity in aging mice [49]; and nicotinamide riboside was found to protect against ROS to extend lifespan in *C. elegans* [50]. However, in our study, no association between nicotinamide intake and protein carbonyl content was observed. Except for a recent randomized controlled trial combining nicotinamide adenine dinucleotide (NADH) and co-enzyme Q10 that reduced fatigue in CFS patients [51], so far, evidence on NAD^+^ targeting interventions against patient-reported outcomes is scarce. More translational research is needed to bridge the gap between animal studies and human populations. Additionally, it is still under debate whether inflammation and oxidative stress are the underlying mechanisms that lead to fatigue and mental complaints. Emerging evidence is also inconsistent, and there are only a few studies involving cancer survivors. Some cross-sectional studies have found associations between certain cytokines, oxidative stress markers (e.g., protein carbonyl contents, lipid peroxidation markers), and fatigue or depression [18,19,41], while another study did not [40]. Recently, an observational study suggested that 45% of the association between adhering to the WCRF dietary lifestyle score and fatigue was mediated by inflammatory markers [52]. Our study did not observe significant associations between protein carbonyl contents and patient-reported outcomes, which may be due to the relatively long years of survival (up to 2–10 years) of participants. In these survivors, the anti-oxidative system may already have been activated to counteract the stress, or this may be due to the relatively small sample size. The degradation of samples over long storage time (8 years) may also have had an effect on serum protein carbonyl contents. We did not find any significant association between nicotinamide intake and hand-grip strength, which is an indicator of muscle strength. This maybe also due to the small sample size of our study, or that other NAD^+^ generating precursors, e.g., nicotinamide riboside may be more relevant in terms of maintaining muscle strength.

Recent study has demonstrated that most tissues rely on nicotinamide for NAD^+^ synthesis [53]. From our data, we did not find any association between nicotinamide intake and serum NAD^+^ levels. In other words, a higher level of nicotinamide intake was not correlated with higher serum NAD^+^ levels. However, the serum NAD^+^ levels we measured were lower than those reported recently [54]. This difference may be the result of the long period storage of the serum in our study (8 years) versus the other study (half a year) [54]. We included “sample storage time” as a covariate into the final regression model, and yet no association between NAD^+^ levels and outcomes was observed, which indicates that when investigating the relation between NAD^+^-status and physical and mental outcomes of cancer survivors, serum samples should be used that are stored for a shorter period.

Currently, no specific guidelines are available indicating which nicotinamide intake levels are suggested among CRC survivors. Thus, even though according to our findings, a higher level of nicotinamide intake seems to be beneficial, CRC survivors are advised to follow dietary guidelines for general populations (i.e., for adult women without pregnancy nor breastfeeding and men, 14 and 16 mg/d, respectively). However, we doubt whether this may be sufficient to counteract an increased activity of NAD^+^-degrading enzymes, for instance poly (ADP-ribose) polymerase 1 (PARP1) and CD38, due to inflammatory conditions induced by cancer cell metabolism itself as well as by treatment [55,56,57]. In this study, almost 50% of the female CRC survivors did not meet the Dutch RDA level (13 mg/d) [16]. Therefore, it is advised to include an assessment of nicotinamide intake in dietary assessments and interventions for cancer survivors.

One of the strengths of our study is that we selected a relatively rigorous criterion to adjust for potential confounders, considering factors such as MVPA levels, energy intake, and intake of supplements, which allowed for a comprehensive understanding of associations between nicotinamide intake and patient-reported outcomes. However, some limitations must also be considered. First, similar to other cross-sectional studies, we cannot draw conclusions about causality. Participants reporting higher levels of fatigue and anxiety might have a poorer appetite or fail to follow an adequate and balanced diet, which may lead to a lower level of nicotinamide intake. However, after adjusting for daily total energy intake and the use of supplements, the associations we observed remained significant and unchanged. In addition, we calculated nicotinamide intake only, without considering the contribution from dietary L-tryptophan that is used for de novo NAD^+^ synthesis. Although it is generally assumed that 60 mg of tryptophan is equivalent to 1 mg of nicotinamide [58], the tryptophan intake of CRC survivors is not known. Based on intake data from a recent study including 40 Dutch hemodialysis patients, it is considered likely that the tryptophan intake of Dutch adults is meeting the requirements, since this study reported a mean dietary tryptophan intake of 909 ± 235 mg/d [59]. NAD^+^ can be synthesized from tryptophan by the kynurenine pathway [60], and it can be speculated that a low dietary intake of nicotinamide may lead to an increased production of NAD^+^, which may lead to the accumulation of metabolites produced by the kynurenine pathway. Recently, plasma kynurenine metabolites were found to possibly mediate an inflammation-associated depressive symptom profile in patients with depression syndromes [61]. Secondly, the cross-sectional design with a relatively limited sample size does prohibit a definitive or causal conclusion about nicotinamide intake and patient-reported outcomes. Further prospective follow-up and intervention studies that include a reliable estimation of NAD^+^ status are warranted to corroborate these findings.

## 5. Conclusions

In conclusion, our findings indicate that a higher intake of dietary nicotinamide is inversely associated with fatigue and anxiety symptoms reported by long-term CRC survivors, 2 to 10 years post-diagnosis. CRC survivors and health care professionals should be aware that having a balanced diet is of importance with respect to a sufficient nicotinamide intake. Further prospective and intervention studies are warranted to have a better understanding on the potential beneficial effects of nicotinamide and other NAD^+^ precursors on patient-reported outcomes such as fatigue, depression, and anxiety.

## Figures and Tables

**Table 1:** Socio-demographic and clinical characteristics of colorectal cancer survivors (*n* = 145), diagnosed with stage I–III colorectal cancer at Maastricht University Medical Center (2002–2010).

	Male (*n* = 91)	Female (*n* = 54)	Total Population (*n* = 145)
Age, mean (SD)	70.0 (8.0)	70.0 (10.0)	70.0 (9.0)
BMI, *n* (%)			
<25 kg/m^2^	21 (23.1)	17 (31.5)	38 (26.2)
≥25 kg/m^2^	70 (76.9)	37 (68.5)	107 (73.8)
Smoking status, *n* (%)			
Former or never	79 (86.8)	50 (92.6)	127 (87.5)
Current	12 (13.2)	4 (7.4)	18 (12.5)
Number of comorbidities, *n* (%)			
0	25 (27.5)	11 (20.4)	35 (24.1)
1	22 (24.2)	12 (22.2)	34 (23.4)
≥2	44 (48.4)	31 (57.4)	76 (52.4)
Cancer stage, *n*(%)			
I	23 (23.5)	16 (29.6)	49 (33.8)
II	31 (34.1)	21 (38.9)	52 (35.9)
III	30 (33.0)	17 (31.5)	47 (32.4)
Received chemotherapy, *n* (%)			
Yes	48 (52.7)	27 (50)	75 (51.7)
Received radiotherapy, *n* (%)			
Yes	41 (45.1)	13 (24.1)	54 (37.2)
Received surgery, *n* (%)			
Yes	86 (94.5)	53 (98.1)	139 (95.9)
Years since diagnosis, median (IQR)	6.0 (3.0)	6.0 (3.0)	6.0 (3.0)
Hours per week of MVPA, median (IQR)	9.9 (12.9)	7.0 (8.3)	8.6 (10.3)
Dietary intake			
Energy (kcal/d), median (IQR)	2157.6 (494.0)	1578.9 (382.2)	1930.4 (654.0)
Nicotinamide (mg/d), median (IQR)	19.1 (7.5)	14.4 (6.3)	17.3 (8.0)
Supplement use (yes/no), *n* (%)			
Yes	38 (41.8)	26 (48.1)	64 (44.1)
Serum protein carbonyl contents (nmol/mg protein), mean (SD)	32.7 (19.6)	38.5 (23.3)	34.9 (21.2)
NAD^+^(nmol/L)*,* mean *(SD)*	1572.4 (731.5)	1462.6 (566.4)	1531.3 (674.4)

**Table 2:** Associations of daily nicotinamide intake with hand-grip strength, emotional and cognitive functioning, fatigue, anxiety, and depression.

	Univariate		Model I ^a^		Model II ^b^	
	β ^c^	95% CI	β ^c^	95% CI	β ^c^	95% CI
Hand-grip strength	**19.13**	**14.53, 23.74**	**4.81**	**0.87, 8.75**	2.30	−2.41, 7.02
Emotional functioning	**10.90**	**3.37, 18.43**	**11.77**	**2.53, 21.01**	10.31	−0.65, 21.26
Cognitive functioning	6.63	−1.99, 15.25	6.77	−3.91, 17.45	8.04	−4.50, 20.58
Fatigue						
According to EORTC	**−17.25**	**−26.43, −8.01**	**−19.64**	**−31.03, −8.25**	**−14.85**	**−28.14, −1.56**
According to CIS total	**−16.45**	**−28.44, −4.45**	**−19.91**	**−34.72, −5.11**	**−17.52**	**−34.54, −0.51**
CIS subjective fatigue	**−9.47**	**−15.23, −3.70**	**−11.63**	**−18.74, −4.53**	**−10.47**	**−18.70, −2.25**
CIS activity	**−3.19**	**−5.00, −0.24**	**−3.88**	**−6.79, −1.00**	−2.93	−6.20, 0.33
CIS motivation	**−3.19**	**−5.75, −0.63**	**−3.36**	**−6.54, −0.18**	−2.19	−5.79, 1.42
CIS concentration	−1.28	−4.62, 2.07	−0.83	−5.00, 3.33	−0.86	−5.82, 4.11
HADS total (distress)	**−3.13**	**−5.81, −0.45**	**−4.38**	**−7.67, −1.09**	**−4.69**	**−8.55, −0.83**
HADS depression	−1.17	−2.69, 0.35	**−2.04**	**−3.92, −0.16**	−2.12	−4.32, 0.07
HADS anxiety	**−2.11**	**−3.68, −0.55**	**−2.53**	**−4.42, −0.65**	**−2.55**	**−4.79, −0.32**

^a^ Model adjusted by age, gender, and BMI; ^b^ Model fully adjusted by daily average energy, number of comorbidities, received chemotherapy (yes/no), hours of per week MVPA, years since diagnosis, and supplement usage (yes/no); ^c^ To interpret the beta coefficient of the regression line, since a natural log transformation was done on the independent variable, the beta coefficient indicates that a 10% increase in nicotinamide intake leads to a 2.30 β units’ changes of outcomes. Significantly associations were denoted with bold.

**Table 3:** Results of subgroup analyses of daily nicotinamide intake dosage with hand-grip strength, emotional and cognitive functioning, fatigue, anxiety, and depression.

	^b^ G2 vs. G1 (Ref)				*P*-Interaction
	Male (*n* = 70)		Female (*n* = 29)		
	β	95% CI	β	95% CI	
Hand-grip strength ^a^	3.35	−1.23, 7.93	−0.08	−3.40, 3.33	<0.001
Emotional functioning ^a^	**11.21**	**1.64, 20.79**	−1.42	−12.35, 9.50	0.002
Cognitive functioning ^a^	−0.71	−11.19, 9.77	−4.31	−18.28, 9.67	0.924
Fatigue					
According to EORTC ^a^	**−10.43**	**−20.93, −0.17**	−2.60	−18.14, 12.93	0.005
According to CIS (total) ^a^	**−8.97**	**−23.14, −0.20**	−0.85	−20.41, 18.72	0.270
CIS subjective fatigue ^a^	**−6.35**	**−13.57, −0.18**	−0.73	−9.65, 8.20	0.076
CIS activity ^a^	−0.91	−3.67, 1.85	0.68	−2.81, 4.18	0.789
CIS motivation ^a^	−1.08	−4.18, 2.03	−1.96	−5.88, 1.96	0.207
CIS concentration ^a^	−1.60	−6.00, 2.78	1.75	−3.16, 6.67	0.614
HADS total (distress) ^a^	**−3.50**	**−6.94, −0.50**	−2.08	−5.60, 1.45	0.050
HADS depression ^a^	−1.57	−3.61, 0.48	−0.82	−2.60, 0.96	0.579
HADS anxiety ^a^	**−2.31**	**−4.31, −0.30**	−1.14	−3.35, 1.07	0.005

^a^ Model fully adjusted by age, BMI, daily average energy, number of comorbidities, received chemotherapy (yes/no), hours of per week MVPA, years since diagnosis and supplement usage (yes/no); ^b^ Daily dietary nicotinamide intake were dichotomized into two groups, G1 (≤14 mg/d for female, *n* = 25, ≤16 mg/d for male, *n* = 21; as reference), G2 (>14 mg/d for female and >16 mg/d for male). β was calculated by setting the G1 as reference. Significantly associations were denoted with bold.

## Data Availability

Data described in the manuscript, code book, and analytic code will be made available upon request pending (e.g., application and approval, payment, other). Requests for data of the EnCoRe study can be sent to Martijn Bours, Department of Epidemiology, GROW-School for Oncology and Developmental Biology, Maastricht University, The Netherlands (e-mail: m.bours@maastrichtuniversity.nl).

## Supplementary Materials

The following are available online at https://www.mdpi.com/article/10.3390/nu13113707/s1, Table S1: Associations of serum NAD+ levels with hand-grip strength, emotional and cognitive functioning, fatigue, anxiety, and depression. Table S2: Associations of serum protein carbonyl contents with hand-grip strength, emotional and cognitive functioning, fatigue, anxiety, and depression. Table S3: Dose-response relationships between daily nicotinamide intake and hand-grip strength, emotional and cognitive functioning, fatigue, anxiety, and depression.
