# Supplementary material for: Cross-Sectional Associations between Dietary Daily Nicotinamide Intake and Patient-Reported Outcomes in Colorectal Cancer Survivors, 2 to 10 Years Post-Diagnosis
Source: Nutrients. 2021 Oct 21;13(11):3707. doi: 10.3390/nu13113707 (PMC8624000; doi:10.3390/nu13113707)
Supplement: Supplementary file 1 [file nutrients-13-03707-s001.zip › nutrients-1392538-supplementary.pdf]

Supplementary

Table S1. Dose-response relationships between daily nicotinamide intake and hand-drip strength, emotional and cognitive functioning, fatigue, anxiety, and depression.

	G1 [b] (*n*=37)		G2 [b] (*n*=37)		G3 [b] (*n*=61)		P-trend
	β	95% CI	β	95% CI	β	95% CI	
Hand-drip strength			1.98	−1.13, 5.08	3.07	−0.89, 7.03	0.12
Emotional functioning		Ref.	5.38	−1.93, 12.69	9.37	0.06, 18.68	0.04
Cognitive functioning		Ref.	−2.04	−10.49, 6.41	−2.50	−13.27, 8.26	0.70
Fatigue		Ref.					
According to EORTC			−6.75	−15.63, 2.13	−9.00	−20.30, 2.33	0.05
According to CIS		Ref.	−6.97	−18.40, 4.47	−9.45	−23.80, 4.97	0.06
total		Ref.					
CIS Subjective		Ref.	−4.03	−9.54, 1.49	−6.70	−13.72, 0.32	0.06
fatigue		Ref.					
CIS Activity		Ref.	−0.11	−2.30, 2.08	−1.30	−4.07, 1.47	0.37
CIS motivation		Ref.	−1.67	−4.06, 0.73	−1.69	−4.74, 1.36	0.25
CIS concentration		Ref.	−0.30	−3.60, 3.01	0.51	−3.70, 4.72	0.83
HADS total (distress)		Ref.	−4.38	−7.67, −1.09	−4.69	−8.55, −0.83	0.02
HADS depression		Ref.	−2.04	−3.92, −0.16	−2.12	−4.32, 0.07	0.06
HADS anxiety			−2.53	−4.42, −0.65	−2.55	−4.79, −0.32	0.02

[a] Model adjusted by daily average energy, number of comorbidities, received chemotherapy (yes/no), hours of per week MVPA, years since diagnosis and supplement usage (yes/no); [b] nicotinamide intake was dichotomized into three groups, G1 (below RDA, for male participants it is <16 mg/d, while for female participants it is <14 mg/d) , G2 (between RDA and 1.25 times RDA, for male and female participants, 16–20 mg/d and 14–17.5 mg/d, respectively), and G3 (above 1.25 times of RDA).

Table S2. Associations of serum NAD^+ levels with hand-drip strength, emotional and cognitive functioning, fatigue, anxiety, and depression.

	Univariate		Model I [a]		Model II [b]	
	β [c]	95% CI	β [c]	95% CI	β [c]	95% CI
Hand-grip strength	−0.17	−2.30, 1.95	0.02	−1.23, 1.30	0.09	−1.20, 1.37
Emotional functioning	−0.52	−3.52, 2.49	−0.53	−3.51, 2.46	0.02	−3.03, 3.06
Cognitive functioning	−0.01	−0.10, 0.07	−0.01	−0.10, 0.08	0.00	−0.08, 0.10
Fatigue						
According to EORTC	0.18	−3.54, 3.89	0.14	−3.58, 3.87	−0.81	−4.48, 2.85
According to CIS total	1.57	−3.17, 6.32	1.53	−3.23, 6.28	−0.37	−5.03,4.29
CIS Subjective fatigue	0.88	−1.43,3.19	0.85	−1.47, 3.16	0.14	−2.15, 2.42
CIS Activity	0.32	−0.62, 1.26	0.31	−0.63, 1.25	−0.20	−1.08, 0.69
CIS motivation	0.22	−0.79, 1.23	0.20	−0.81, 1.22	0.01	−0.96, 0.98
CIS concentration	−0.04	−1.31, 1.23	−0.05	−1.34, 1.23	−0.45	−1.79, 0.89
HADS total (distress)	−0.10	−1.17, 0.96	−0.11	−1.17, 0.95	−0.39	−1.46, 0.68
HADS depression	0.07	−0.53, 0.66	0.07	−0.53, 0.67	−0.09	−0.70, 0.52
HADS anxiety	−0.66	−0.68, 0.55	−0.07	−0.67, 0.53	−0.13	−0.75, 0.49

[a] Model adjusted by age, gender and BMI; [b] Model fully adjusted by daily average energy, number of comorbidities, received chemotherapy (yes/no), hours of per week MVPA, years since diagnosis and supplement usage (yes/no); [c] To interpret the beta

coefficient of the regression line, since a natural log transformation was done on the independent variable, the beta coefficient indicates that a 10% increase of nicotinamide intake lead to a 2.30β units' changes of outcomes.

Table S3. Associations of serum protein carbonyl contents with hand-drip strength, emotional and cognitive functioning, fatigue, anxiety, and depression.

	Univariate		Model I [a]		Model II [b]	
	β [c]	95% CI	β [c]	95% CI	β [c]	95% CI
Hand-drip strength	−0.39	−2.47, 1.59	0.68	−0.58, 1.95	0.67	−0.60, 1.93
Emotional functioning	−0.02	−3.00, 2.94	0.73	−2.29, 3.75	0.54	−2.46, 3.54
Cognitive functioning	0.04	−0.04, 0.13	0.04	−0.05, 0.13	0.04	−0.05, 0.13
Fatigue						
According to EORTC	−0.84	−4.50, 2.82	−1.37	−5.14, 2.40	−1.34	−4.95, 2.26
According to CIS total	0.85	−3.82, 5.52	1.39	−3.45, 6.22	1.13	−3.47, 5.73
CIS Subjective fatigue	0.73	−1.55, 3.00	0.80	−1.55, 3.14	0.72	−1.53, 2.96
CIS Activity	0.15	−0.78, 1.26	0.34	−0.61, 1.30	0.39	−0.48, 1.26
CIS motivation	−0.17	−1.16, 0.83	−0.15	−1.18, 0.88	−0.12	−1.08, 0.83
CIS concentration	0.06	−1.31, 1.23	0.03	−1.30, 1.33	−0.12	−1.43, 1.21
HADS total (distress)	0.48	−0.54, 1.51	0.30	−0.77, 1.33	0.41	−0.63, 1.44
HADS depression	0.00	−0.57, 0.58	0.05	−0.54, 0.65	0.06	−0.53, 0.64
HADS anxiety	0.38	−0.23, 0.99	0.19	−0.42, 0.80	0.27	−0.34, 0.87

[a] Model adjusted by age, gender and BMI; [b] Model fully adjusted by daily average energy, number of comorbidities, received chemotherapy (yes/no), hours of per week MVPA, years since diagnosis and supplement usage (yes/no); [c] To interpret the beta coefficient of the regression line, since a natural log transformation was done on the independent variable, the beta coefficient indicates that a 10% increase of nicotinamide intake lead to a 2.30β units' changes of outcomes.
